# WSES Guidelines for the management of acute left sided colonic diverticulitis in the emergency setting

**DOI:** 10.1186/s13017-016-0095-0

**Published:** 2016-07-29

**Authors:** Massimo Sartelli, Fausto Catena, Luca Ansaloni, Federico Coccolini, Ewen A. Griffiths, Fikri M. Abu-Zidan, Salomone Di Saverio, Jan Ulrych, Yoram Kluger, Ofir Ben-Ishay, Frederick A. Moore, Rao R. Ivatury, Raul Coimbra, Andrew B. Peitzman, Ari Leppaniemi, Gustavo P. Fraga, Ronald V. Maier, Osvaldo Chiara, Jeffry Kashuk, Boris Sakakushev, Dieter G. Weber, Rifat Latifi, Walter Biffl, Miklosh Bala, Aleksandar Karamarkovic, Kenji Inaba, Carlos A. Ordonez, Andreas Hecker, Goran Augustin, Zaza Demetrashvili, Renato Bessa Melo, Sanjay Marwah, Sanoop K. Zachariah, Vishal G. Shelat, Michael McFarlane, Miran Rems, Carlos Augusto Gomes, Mario Paulo Faro, Gerson Alves Pereira Júnior, Ionut Negoi, Yunfeng Cui, Norio Sato, Andras Vereczkei, Giovanni Bellanova, Arianna Birindelli, Isidoro Di Carlo, Kenneth Y Kok, Mahir Gachabayov, Georgios Gkiokas, Konstantinos Bouliaris, Elif Çolak, Arda Isik, Daniel Rios-Cruz, Rodolfo Soto, Ernest E. Moore

**Affiliations:** 1 Department of Surgery, Macerata Hospital, Via Santa Lucia 2, 62019 Macerata, Italy; 2 Department of Surgery, Maggiore Hospital, Parma, Italy; 3 General Surgery Department, Papa Giovanni XXIII Hospital, Bergamo, Italy; 4 Department of Surgery, “Infermi” Hospital, Rimini, Italy; 5 General and Upper GI Surgery, Queen Elizabeth Hospital, Birmingham, UK; 6 Department of Surgery, College of Medicine and Health Sciences, UAE University, Al-Ain, United Arab Emirates; 7 Department of Surgery, Maggiore Hospital, Bologna, Italy; 8 1st Department of Surgery - Department of Abdominal, Thoracic Surgery and Traumatology, General University Hospital, Prague, Czech Republic; 9 Department of General Surgery, Division of Surgery, Rambam Health Care Campus, Haifa, Israel; 10 Department of Surgery, Division of Acute Care Surgery, and Center for Sepsis and Critical Illness Research, University of Florida College of Medicine, Gainesville, FL USA; 11 Department of Surgery, Virginia Commonwealth University, Richmond, VA USA; 12 Department of Surgery, UC San Diego Medical Center, San Diego, USA; 13 Department of Surgery, University of Pittsburgh School of Medicine, Pittsburgh, USA; 14 Abdominal Center, University Hospital Meilahti, Helsinki, Finland; 15 Division of Trauma Surgery, Department of Surgery, School of Medical Sciences, University of Campinas (Unicamp), Campinas, SP Brazil; 16 Department of Surgery, University of Washington, Seattle, WA USA; 17 Emergency Department, Niguarda Ca’Granda Hospital, Milan, Italy; 18 Assia Medical Group, Assuta Medical Center, Tel Aviv, Israel; 19 First Clinic of General Surgery, University Hospital/UMBAL/St George Plovdiv, Plovdiv, Bulgaria; 20 Department of Traumatology, John Hunter Hospital, Newcastle, NSW Australia; 21 Department of Surgery, Trauma Research Institute, University of Arizona, Tucson, AZ USA; 22 Department of Surgery, University of Colorado, Denver Health Medical Center, Denver, CO USA; 23 Trauma and Acute Care Surgery Unit, Hadassah Hebrew University Medical Center, Jerusalem, Israel; 24 Clinic for Emergency Surgery, Faculty of Medicine, University of Belgrade, Belgrade, Serbia; 25 Department of Surgery, Division of Acute Care Surgery and Surgical Critical Care, Los Angeles County and University of Southern California Medical Center, Los Angeles, CA USA; 26 Department of Surgery, Fundación Valle del Lili, Hospital Universitario del Valle, Universidad del Valle, Cali, Colombia; 27 Department of General and Thoracic Surgery, University Hospital Giessen, Giessen, Germany; 28 Department of Surgery, University Hospital Center Zagreb and School of Medicine, University of Zagreb, Zagreb, Croatia; 29 Department of Surgery, Tbilisi State Medical University, Kipshidze Central University Hospital, Tbilisi, Georgia; 30 Department of General Surgery, Centro Hospitalar São João, Faculdade de Medicina da Universidade do Porto, Porto, Portugal; 31 Department of Surgery, Pt BDS Post-graduate Institute of Medical Sciences, Rohtak, India; 32 Department of Surgery, Mosc Medical College, Kolenchery Cochin, India; 33 Department of General Surgery, Tan Tock Seng Hospital, Tan Tock Seng, Singapore, Singapore; 34 Department of Surgery, Radiology, Anaesthetics and Intensive Care, University Hospital of the West Indies, Kingston, Jamaica; 35 Surgical Department, General Hospital Jesenice, Jesenice, Slovenia; 36 Federal University of Juiz de Fora (UFJF) AND Faculdade de Ciências Médicas e da Saúde de Juiz de Fora (SUPREMA), Juiz de Fora, MG Brazil; 37 Department of General Surgery, Trauma and Emergency Surgery Division, ABC Medical School, Santo André, SP Brazil; 38 Emergency Surgery and Trauma Unit, Department of Surgery, University of Ribeirão Preto, Ribeirão Preto, Brazil; 39 Emergency Hospital of Bucharest, University of Medicine and Pharmacy Carol Davila Bucharest, Bucharest, Romania; 40 Department of Surgery, Tianjin Nankai Hospital, Nankai Clinical School of Medicine, Tianjin Medical University, Tianjin, China; 41 Department of Primary Care & Emergency Medicine, Kyoto University Graduate School of Medicine, Kyoto, Japan; 42 Department of Surgery, Medical School University of Pécs, Pécs, Hungary; 43 Department of Surgery, S.S. Annunziata Hospital, Taranto, Italy; 44 Department of Surgical Sciences, Organs Transplantation and Advanced Technologies, “G.F. Ingrassia” University of Catania, Cannizzaro Hospital, Catania, Italy; 45 Department of Surgery, The Brunei Cancer Centre, Jerudong Park, Brunei; 46 Department of Surgery, Clinical Hospital of Emergency Medicine, Vladimir City, Russian Federation; 47 2nd Department of Surgery, Aretaieion University Hospital, National and Kapodistrian University of Athens, Athens, Greece; 48 Department of General Surgery, University Hospital of Larissa, Larissa, Greece; 49 Department of Surgery, Samsun Education and Research Hospital, Samsun, Turkey; 50 Department of Surgery, Mengucek Gazi Training Research Hospital, Erzincan, Turkey; 51 Department of Surgery, Hospital de Alta Especialidad de Veracruz, Veracruz, Mexico; 52 Department of Emergency Surgery and Critical Care, Centro Medico Imbanaco, Cali, Colombia

## Abstract

Acute left sided colonic diverticulitis is one of the most common clinical conditions encountered by surgeons in acute setting. A World Society of Emergency Surgery (WSES) Consensus Conference on acute diverticulitis was held during the 3rd World Congress of the WSES in Jerusalem, Israel, on July 7th, 2015. During this consensus conference the guidelines for the management of acute left sided colonic diverticulitis in the emergency setting were presented and discussed. This document represents the executive summary of the final guidelines approved by the consensus conference.

## Background

Acute left sided colonic diverticulosis is common in Western countries, however its prevalence is increasing throughout the world, probably because of changes in lifestyle [1]. Although left sided colonic diverticulosis is more common amongst elderly patients, a dramatic rise of its incidence has been seen in the younger age groups in recent years [2]. Data from Western populations suggest that up to one fifth of patients with acute diverticulitis are under the age of 50 years of age [3–5]. Recent evidence suggests that lifetime risk of developing acute left sided colonic diverticulitis (ALCD) is only about 4 % among patients with diverticulosis [6].

ALCD is a common problem encountered by surgeons in the acute setting. It encompasses a variety of conditions, ranging from localized diverticular inflammation to perforation and fecal peritonitis. Daily decisions in the diagnosis and treatment of acute diverticulitis generally depend on clinicians’ personal preferences rather than evidence-based medicine. There is generally a lack of well conducted randomized clinical trials in ALCD and a large amount of evidence in the literature is low quality and conflicting.

## Methods

A World Society of Emergency Surgery (WSES) working group published in 2015 a proposal for a new CT based classification for ALCD [7]. This has been extended and developed into guidelines for the management of acute diverticulitis in an emergency setting. A literature search, using the PubMed database, was performed without restriction of time or type of manuscript. The search was limited to English language publications. The final grade of recommendation was performed by using the Grades of Recommendation, Assessment, Development, and Evaluation (GRADE) system (Table 1) [8, 9].

**Table 1:** Grading of recommendations from Guyatt and colleagues [8, 9]

rade of recommendation	Clarity of risk/benefit	Quality of supporting evidence	Implications
1A			
Strong recommendation, high-quality evidence	Benefits clearly outweigh risk and burdens, or vice versa	RCTs without important limitations or overwhelming evidence from observational studies	Strong recommendation, applies to most patients in most circumstances without reservation
1B			
Strong recommendation, moderate-quality evidence	Benefits clearly outweigh risk and burdens, or vice versa	RCTs with important limitations (inconsistent results, methodological flaws, indirect analyses or imprecise conclusions) or exceptionally strong evidence from observational studies	Strong recommendation, applies to most patients in most circumstances without reservation
1C			
Strong recommendation, low-quality or very low-quality evidence	Benefits clearly outweigh risk and burdens, or vice versa	Observational studies or case series	Strong recommendation but subject to change when higher quality evidence becomes available
2A			
Weak recommendation, high-quality evidence	Benefits closely balanced with risks and burden	RCTs without important limitations or overwhelming evidence from observational studies	Weak recommendation, best action may differ depending on the patient, treatment circumstances, or social values
2B			
Weak recommendation, moderate-quality evidence	Benefits closely balanced with risks and burden	RCTs with important limitations (inconsistent results, methodological flaws, indirect or imprecise) or exceptionally strong evidence from observational studies	Weak recommendation, best action may differ depending on the patient, treatment circumstances, or social values
2C			
Weak recommendation, Low-quality or very low-quality evidence	Uncertainty in the estimates of benefits, risks, and burden; benefits, risk, and burden may be closely balanced	Observational studies or case series	Very weak recommendation; alternative treatments may be equally reasonable and merit consideration

A World Society of Emergency Surgery (WSES) Consensus Conference on acute diverticulitis was held during the 3rd World Congress of the WSES in Jerusalem, Israel, on July 7th, 2015. During this consensus conference the guidelines were presented and debated. This document represents the executive summary of the final guidelines approved by the consensus conference.

## Results

### Classification systems

ALCD ranges in severity from uncomplicated inflammatory diverticulitis to complicated diverticulitis (abscess formation or perforation). For the past three decades, the Hinchey classification has been the most commonly used classification for complicated ALCD in international literature [10].

Based on the surgical findings of abscesses and peritonitis, Hinchey et al. classified the severity of acute diverticulitis into four grades:

- Stage 1 Pericolic abscess
- Stage 2 Pelvic, intra-abdominal, or retroperitoneal abscess
- Stage 3 Generalized purulent peritonitis
- Stage 4 Generalized fecal peritonitis

The management of ALCD has recently changed dramatically in recent years, due to better radiological imaging and availability of non-surgical treatment options. Computer tomography (CT) imaging has become a primary diagnostic tool in the diagnosis and staging of patients with acute diverticulitis and more detailed information provided by CT scans led to several modifications of the Hinchey classification [4, 11–17]. For example, in 1989 Neff et al. presented a new classification based on CT findings. It consisted of five stages, ranging from radiological diagnosis of uncomplicated AD (Stage 0) to pneumoperitoneum with abundant free liquid (Stage 4) [11]:

- Stage 0 Uncomplicated diverticulitis; Diverticula, thickening of the wall, increased density of the pericolic fat
- Stage 1 Locally complicated with local abscess
- Stage 2 Complicated with pelvic abscess;
- Stage 3 Complicated with distant abscess
- Stage 4 Complicated with other distant complications

In 1997, Sher et al. [12] introduced a modification of Hinchey classification. This classification divided abscesses into pericolic abscesses (stage I), distant abscesses amendable for percutaneous drainage (stage IIa), and complex abscesses associated with a possible fistula (stage IIb). This classification implied the use of new treatment strategies, such as CT-guided percutaneous drainage of abscesses.

In 2002 Ambrosetti et al. [13] classified diverticulitis into severe or moderate disease. In this classification, the CT scan determined the grade of severity guiding the physician in the treatment of acute complications. Moderate diverticulitis was defined by wall thickening of ≥ 5 mm and signs of inflammation of pericolic fat. Severe diverticulitis was defined by wall thickening accompanied by abscess formation, extraluminal air or extraluminal contrast leak:

- Moderate diverticulitis Localized sigmoid wall thickening
- Pericolic fat stranding
- Severe diverticulitisAbscess
- Extraluminal air
- Extraluminal contrast

In 2005 Kaiser et al. [14] modified Hinchey classification according to specific CT findings:

- Stage 0 mild clinical diverticulitis
- Stage 1a confined pericolic inflammation,
- Stage 1b confined pericolic abscess
- Stage 2 pelvic or distant intra-abdominal abscess
- Stage 3 generalized purulent peritonitis
- Stage 4 fecal peritonitis at presentation.

In 2013 Mora Lopez et al. proposed [15] a modification of the previous Neff classification dividing Neff stage 1 into stage 1 a (localized pneumoperitoneum in the form of air bubbles) and 1 b abscess (<4 cm):

- Stage 0 Uncomplicated diverticulitis. Diverticula, thickening of the wall, increased density of the pericolic fat
- Stage 1 Locally complicated diverticulitis
- Stage 1a Localized pneumoperitoneum in the form of air bubbles
- Stage 1b Abscess (<4 cm)
- Stage 2 Complicated diverticulitis with pelvic abscess. Abscess > 4 cm in pelvis
- Stage 3 Complicated diverticulitis with distant abscess. Abscess in abdominal cavity (outside pelvis)
- Stage 4 Complicated diverticulitis with other distant complications. Abundant pneumoperitoneum and/or intra-abdominal free liquid

Recently Sallinen at al. [16] published an interesting retrospective study of patients treated for diverticulitis, setting the stage for the treatment of acute diverticulitis based on clinical, radiologic and physiologic parameters. They included 5 stages:

- Stage 1 Uncomplicated diverticulitis
- Stage 2 Complicated diverticulitis with small abscess (<6 cm)
- Stage 3 Complicated diverticulitis with large abscess (≥6 cm) or distant intraperitoneal or retroperitoneal air
- Stage 4 Generalized peritonitis without organ dysfunction
- Stage 5 Generalized peritonitis with organ dysfunction

Finally a proposal for a CT guided classification of left colon acute diverticulitis was published in 2015 by the WSES acute diverticulitis working group [7]. It is a simple classification system of acute diverticulitis based on CT scan findings. It may guide clinicians in the management of acute diverticulitis and may be universally accepted for day to day practice. The WSES classification divides acute diverticulitis into 2 groups: uncomplicated and complicated.

In the event of uncomplicated acute diverticulitis, the infection does not extend to the peritoneum. In the event of complicated acute diverticulitis, the infectious process proceeds beyond the colon. Complicated acute diverticulitis is divided into 4 stages, based on the extension of the infectious process:

#### Uncomplicated

- Stage 0 Diverticula, thickening of the colonic wall or increased density of the pericolic fat
- Complicated
- Stage 1a Pericolic air bubbles or little pericolic fluid without abscess (within 5 cm from inflamed bowel segment)
- Stage 1b Abscess ≤ 4 cm
- Stage 2a Abscess > 4 cm
- Stage 2b Distant air (>5 cm from inflamed bowel segment)
- Stage 3 Diffuse fluid without distant free air (no hole in colon)
- Stage 4 Diffuse fluid with distant free air (persistent hole in colon)

### Diagnosis

1. An accurate assessment of the patients using clinical signs, laboratory inflammation markers and radiological findings is recommended to identify the best treatment for each patient with ALCD (Recommendation 1 C).
2. The clinical diagnosis of ALCD alone is not sufficiently accurate for patients with suspected diverticulitis (Recommendation 1 C).
3. Pain in the lower left abdomen on physical examination and C-reactive protein (CRP) 50 mg/l or more suggests a diagnosis of ALCD (Recommendation 1 C).

Clinical findings of patients having ALCD include acute pain or tenderness in the left lower quadrant which may be associated with increased inflammatory markers including C-reactive protein (CRP) and white blood cell count (WBC). Clinical diagnosis of ALCD usually lacks accuracy. In a prospective analysis [17] conducted on 802 consecutive patients that presented with abdominal pain to the emergency department, positive and negative predictive values of clinical diagnosis were 0.65 and 0.98 respectively. Additional cross-sectional imaging had a positive and negative predictive value of more 0.95 and 0.99 respectively. Additional radiology examinations improved the diagnostic accuracy in 37 % of the patients, but changed the management in only 7 %. Ultrasound and CT had superior diagnostic accuracy, however these examinations rarely changed the initial management proposal.

Using logistic regression analysis, Lameris et al. [18] in 2010, developed a clinical decision rule for diagnosis of diverticulitis, based on 3 criteria: direct tenderness in the left lower quadrant, 2) CRP > 50 mg/l and 3) absence of vomiting. Of 126 clinically suspected patients enrolled in this prospective study, 30 patients had all 3 features (24 %), of whom 29 had a final diagnosis of acute diverticulitis (97 %; 95 % CI: 83 %-99 %). Of the 96 patients without all 3 features, 45 (47 %) did not have diverticulitis. In a quarter of patients with suspected diverticulitis, the diagnosis could be made clinically based on a combination of the three criteria.

Andeweg et al. in 2011 [19], using retrospective data from 287 patients, developed a clinical scoring system for the diagnosis of ALCD that had a diagnostic accuracy of 86 %. It was based on the independent predictors of ALCD and included patients’ age, one or more previous episodes, localization of symptoms in the lower left abdomen, aggravation of pain on movement, the absence of vomiting, localization of abdominal tenderness in the lower left abdomen, and C-reactive protein 50 or more.

CRP has been identified as a useful biomarker of inflammation and it may be useful in the prediction of the clinical severity of acute diverticulitis as demonstrated by several recent studies [20–22]. To investigate the value of C-reactive protein (CRP) and of other laboratory parameters of the patients in the prediction of the clinical severity of acute diverticulitis a retrospective study was published in 2014 [20]. A CRP cutoff value of 170 mg/L significantly discriminated severe from mild diverticulitis (87.5 % sensitivity, 91.1 % specificity, area under the curve 0.942, *P* < 0.00001). The author concluded that CRP is a useful tool in the prediction of the clinical severity of acute diverticulitis. A mild episode is very likely in patients with CRP less than 170 mg/L. Those with higher CRP values have a greater probability of undergoing surgery or radiological drainage.

In another study, the diagnostic value of serological infection markers and body temperature in discriminating complicated from uncomplicated diverticulitis was assessed [21]. A total of 426 patients were included in this study of which 364 (85 %) presented with uncomplicated and 62 (15 %) with complicated diverticulitis. Only CRP was of sufficient diagnostic value (area under the curve 0.715). The median CRP in patients with complicated diverticulitis was significantly higher than in patients with uncomplicated disease (224 mg/l, range 99–284 vs 87 mg/l, range 48–151). Patients with a CRP of 25 mg/l had a 15 % chance of having complicated diverticulitis. This increased from 23 % at a CRP value of 100 mg/l to 47 % for 250 mg/l or higher. The optimal threshold was reached at 175 mg/l with a positive predictive value of 36 %, negative predictive value of 92 %, sensitivity of 61 % and a specificity of 82 %.

Recently Makela et al. [22] published a study which compared the CRP values of 350 patients who presented first time with symptoms of acute diverticulitis with the CT findings and clinical parameters by means of both univariate and multivariate analyses. CRP cut-off value of 149.5 mg/l significantly discriminated acute uncomplicated diverticulitis from complicated diverticulitis (specificity 65 %, sensitivity 85 %, area under the curve 0.811, *p* = 0.0001). In multivariate analysis, a CRP value over 150 mg/l and old age were independent risk factors for acute complicated diverticulitis. The mean CRP value was significantly higher in the patients who died (mean CRP of 207) than in those who survived (mean CRP of 139). In addition, a CRP value over 150 mg/l and free abdominal fluid in CT were independent variables predicting postoperative mortality. The study confirmed that CRP is useful for the predicting the severity of acute diverticulitis on admission. The authors concluded that patients with a CRP value higher than 150 mg/l have an increased risk of complicated diverticulitis and should always undergo a CT examination.

4) Computed tomography (CT) scan of the abdomen and pelvis is indicated for all patients with suspected ALCD. It has high sensitivity and specificity and can assess the severity of diverticulitis guiding clinicians in planning treatment (Recommendation 1 C).
5) Ultrasound (US) may be a useful alternative in the initial evaluation of patients with suspected ALCD. It has wide availability and easy accessibility. It may have satisfactory sensitivity and specificity when performed by an expert operator. A step-up approach with CT performed after an inconclusive or negative US may be a safe approach for patients suspected of acute diverticulitis (Recommendation 1 C).

Radiological imaging techniques that are used for diagnosing acute diverticulitis in the emergency setting are computed tomography (CT) and ultrasound (US). CT imaging is becoming by now the gold standard in the diagnosis and staging of patients with ALCD. CT imaging with intravenous contrast has excellent sensitivity and specificity [23–25]. CT findings in patients with ALCD may include diverticulosis with associated colon wall thickening, fat stranding, phlegmon, extraluminal gas, abscess formation or intra-abdominal diffuse fluid. CT imaging can go beyond accurate diagnosis of ALCD; CT criteria may be also used to determine the grade of severity and may drive treatment planning of patients [7]. US is a real-time dynamic examination with wide availability and easy accessibility [26] and may be useful in diagnosing and managing critically ill patients who cannot be moved to CT. Its limitations include operator-dependency, poor assessment in obese patients, difficulty in the detection of free air and deeply located abscesses [27].

A systematic review and meta-analysis of studies [28] that reported diagnostic accuracy of the clinical diagnosis and diagnostic modalities in patients with suspected diverticulitis was published in 2014. Summary sensitivity estimates for US were 90 % (95 % CI: 76-98 %) versus 95 % (95 % CI: 91-97 %) for CT (*p* = 0.86). Summary specificity estimates for US were 90 % (95 % CI: 86-94 %) versus 96 % (95 % CI: 90-100 %) for CT (*p* = 0.04).

Although CT is the most sensitive imaging investigation for patients with suspected acute diverticulitis, a step-up approach with CT performed after an inconclusive or negative US, has been proposed as safe and alternative approach for patients with suspected acute diverticulitis [28, 29]. Magnetic resonance imaging, which is not constrained by the operator dependency limitation of ultrasound [30, 31], until now is difficult to perform at the emergency department.

### Immunocompromised patients

6) Immunosuppression can increase the complication rate in patients with ALCD. Elective sigmoid resection after an episode of ALCD should be recommended in immunocompromised patients (Recommendation 1 C).

Immunocompromised patients, including patients with kidney failure, organ transplant patients and patients using corticosteroids are at increased risk to have complicated diverticulitis requiring emergency surgery [32–35]. Immunocompromised patients may fail standard, nonoperative treatment. As such, most of these patients require urgent surgical intervention, and this is associated with a significantly higher mortality rate [36].

A recent study by Biondo et al. [37] analyzed the relationship between the different causes of immunosuppression (IMS) and diverticulitis. Immunocompromised patients were divided in 5 groups according to the causes of IMS: group I, chronic corticosteroid therapy; group II, transplant patients; group III, malignant neoplasm disease; group IV, chronic renal failure; group V, others immunosuppressant treatment. The rate of emergency surgery was high (39.3 %). It was needed more frequently in group I. Overall, postoperative mortality was of 31.6 % and recurrence rate after successful nonoperative management occurred in 30 patients (27.8 %).

### Treatment of uncomplicated acute diverticulitis

7) Antimicrobial therapy can be avoided in immunocompetent patients with uncomplicated diverticulitis without systemic manifestations of infection (Recommendation 1 A).
8) If patients need antimicrobial therapy, oral administration may be acceptable (Recommendation 1 B).

The utility of antimicrobial therapy in acute uncomplicated diverticulitis has been a point of controversy in the international medical community. In the last few years several studies demonstrated that antimicrobial treatment was not superior to withholding antibiotic therapy in patients with mild unperforated diverticulitis, in terms of clinical resolution [38].

The current consensus is that uncomplicated diverticulitis may be a self-limiting condition in which local host defenses’ can manage the bacterial inflammation without antibiotics in immunocompetent patients. In this context antibiotics may, therefore, not be necessary in the treatment of uncomplicated disease.

A multi-centre randomized trial involving ten surgical departments in Sweden and one in Iceland recruited 623 patients with computed tomography-verified acute uncomplicated left-sided diverticulitis was published in 2012 by Chabok et al. [39]. Patients were randomized to treatment with (314 patients) or without (309 patients) antibiotics. Antibiotic treatment for acute uncomplicated diverticulitis neither accelerated recovery nor prevented complications or recurrence. It should therefore be reserved for the treatment of complicated diverticulitis.

A recent prospective single-arm study analyzed [40] the safety and efficacy of symptomatic (nonantibiotic) treatment for CT-proven uncomplicated diverticulitis during a 30-day follow-up period. Overall, 161 patients were included in the study, and 153 (95 %) completed the 30-day follow-up. A total of 14 (9 %) patients had pericolic air. Altogether, 140 (87 %) patients were treated as outpatients, and 4 (3 %) of them were admitted to the hospital during the follow-up. None of the patients developed complicated diverticulitis or required surgery, but, 2 days (median) after inclusion, antibiotics were given to 14 (9 %, 6 orally, 8 intravenously) patients.

However the high mortality associated with sepsis, requires clinicians to maintain a high index of clinical suspicion, in the conditions that predispose to sepsis. WSES expert panel suggests antimicrobial therapy covering Gram negative and anaerobes in patients with radiological documented uncomplicated acute diverticulitis associated with systemic manifestations of infection.

An appropriate antimicrobial regimen administered for an adequate duration has minimal impact on the emergence of antimicrobial resistance [41].

If antimicrobial therapy is necessary oral administration of antibiotics may be equally as effective as intravenous administration.

A randomized controlled trial of oral versus intravenous therapy for clinically diagnosed acute uncomplicated diverticulitis was published in 2009 [42]. Oral and intravenous regimens utilizing ciprofloxacin and metronidazole were compared. There were 41 patients in the oral arm and 38 in the IV arm (*n* = 79). No patients had to be converted to intravenous antibiotics from the oral group. There was a complete resolution of symptoms in both groups.

In immunocompromised patients antibiotics with a broader-spectrum should be used. No studies have examined the value of dietary restriction or bed rest [43].

9) Outpatient management is suggested for patients with uncomplicated acute diverticulitis, with no comorbidities. These patients should be clinically monitored as outpatients and re-evaluated within 7 days to assess for resolution of the inflammatory processes. Earlier revaluation is necessary if the clinical condition deteriorates (Recommendation 1 B).

Patients with uncomplicated diverticulitis symptoms without significant comorbidities, who are able to take fluids orally and manage themselves at home, can be treated as outpatients. They should be re-evaluated within 7 days. However if the clinical condition deteriorates, re-evaluation should be carried out earlier. Patients with significant comorbidities and unable to take fluids orally should be treated in hospital with intravenous fluid.

Etzioni et al. [44] in 2010 published a retrospective analysis, demonstrating that outpatient treatment was effective for the vast majority (94 %) of patients suffering from acute diverticulitis. A systematic review on outpatient management of acute uncomplicated diverticulitis was recently published in 2014 [45]. Jackson et al. concluded that current evidence suggested that a more progressive, ambulatory-based approach to the majority of cases of acute uncomplicated diverticulitis was justified. Rodríguez-Cerrillo et al. [46] have recently shown that elderly patients with co-morbidities can be safely treated at home avoiding hospital admission.

The DIVER trial [47] has recently demonstrated that outpatient treatment may be safe and effective in selected patients with uncomplicated acute diverticulitis and can reduces the costs without negatively influencing the quality of life of these patients. This multicenter, randomized controlled trial included patients older than 18 years with acute uncomplicated diverticulitis. All the patients underwent abdominal CT. The first dose of antibiotic was given intravenously to all patients in the emergency department and then patients were either admitted to hospital or discharged. Among a total of 132 patients, four patients in those admitted to hospital and three patients in those discharged to home management developed treatment failure (there was no differences between the groups (*P* = 0.62). The overall health care cost per episode was 3 times less in the outpatient treated group, with significant costs savings of €1124.70 per patient. No differences were observed between the groups in terms of quality of life.

### Treatment of localized complicated diverticulitis

10) Patients with CT findings of pericolic air or small fluid collection should be managed by antimicrobial therapy. (Recommendation 1 C).

High mortality associated with sepsis, requires maintaining a high index of clinical suspicion for deterioration and more aggressive management. WSES expert panel routinely recommends antimicrobial therapy in patients with pericolic air or small fluid collection. CT findings of pericolic air in the form of air bubbles or little pericolic fluid without abscess and distant air indicates a complicated acute diverticulitis and antimicrobial therapy should always recommended [7].

### Treatment of diverticular abscesses

11) Patients with small diverticular abscesses (<4-5 cm) may be treated by antibiotics alone (Recommendation 1 C).
12) Patients with large abscesses (>4–5 cm) can best be treated by percutaneous drainage combined with antibiotic treatment (Recommendation 1 C).
13) Whenever percutaneous drainage of the abscess is not feasible or not available, based on the clinical conditions patients with large abscesses can be initially treated by antibiotic therapy alone. However careful clinical monitoring is mandatory. (Recommendation 1 C).

Approximately 15–20 % of patients admitted with acute diverticulitis have an abscess on CT scan [48]. The size of 3–6 cm has been generally accepted as (all of low level of evidence), to be a reasonable limit between antimicrobial versus percutaneous drainage in the management of diverticular abscesses [48–53]. The size of 4–5 cm may be a reasonable limit between antibiotic treatment alone versus percutaneous drainage combined with antibiotic treatment in the management of diverticular abscesses. Based on the clinical conditions also patients with large abscesses can be initially treated by antibiotic therapy alone. However careful clinical monitoring is mandatory. A CT scan should be repeated if the patient fails to show clinical and laboratory improvement.

A retrospective study assessing the effectiveness of antibiotics as sole initial therapy in patients with large diverticular abscess was published in 2015 by Elagili et al. [54]. Thirty-two patients were treated with antibiotics alone because of either technically impossible percutaneous drainage or surgeon preference, while 114 underwent percutaneous drainage. Urgent surgery was required in 8 patients with persistent symptoms during treatment with antibiotics alone (25 %) and in 21 patients (18 %) after initial percutaneous drainage (*p* = 0.21). Patients treated with antibiotics had a significantly smaller abscess diameter (5.9 vs. 7.1 cm, *p* = 0.001) and shorter interval from initial treatment to sigmoidectomy (mean 50 vs. 80 days, *p* = 0.02). The Charlson comorbidity index, initial treatment failure rates, postoperative mortality, overall morbidity, length of hospital stay during treatments, and overall and permanent stoma rates were comparable in the two groups. Postoperative complications following antibiotics alone were significantly less severe than after percutaneous drainage based on the Clavien-Dindo classification (*p* = 0.04).

In patient displaying an appropriate clinical improvement, drainage catheter can be removed when the output has ceased and the patient has improved clinically. In doubtful cases a fistulogram can be performed with water-soluble contrast via the percutaneous drainage catheter prior to drain removal. If no identifiable cavity remains the catheter should be removed.

14) In patients with diverticular abscesses treated conservatively early colonic evaluation (4–6 weeks) should be planned (Recommendation 1 C).
15) In patients with CT-proven uncomplicated diverticulitis treated conservatively (without other risk factors) early follow-up colonoscopy is not required. Patients aged 50 years or older should participate in colorectal cancer screening programs (Recommendation 1 C).

Colonic localized abscess is an uncommon but possible presentation of colon cancer, and it may mimic complicated diverticular disease [55, 56]. It has been demonstrated that the risk of malignancy after a CT-proven uncomplicated diverticulitis is low and in the absence of other indications, routine colonoscopy may not be necessary. A systematic review investigating the rate of colorectal cancer (CRC) found by colonoscopy after an episode of uncomplicated diverticulitis was published in 2014 [57]. Nine studies met the inclusion criteria and included a total number of 2,490 patients with uncomplicated diverticulitis. Subsequent colonoscopy after an episode of uncomplicated diverticulitis was performed in 1,468 patients (59 %). Seventeen patients were diagnosed with CRC, having a prevalence of 1.16 % (95 % confidence interval 0.72-1.9 % for CRC). Hyperplastic polyps were seen in 156 patients (10.6 %), low-grade adenoma in 90 patients (6.1 %), and advanced adenoma was reported in 32 patients (2.2 %). The results of this review demonstrates that unless colonoscopy is regarded for screening in individuals aged 50 years and older, routine colonoscopy in the absence of other clinical signs of CRC is not required in patients following an episode of acute uncomplicated diverticulitis.

Another systematic review and meta-analysis on the role of routine colonic evaluation after radiologically confirmed acute diverticulitis was published in 2014 [58]. Eleven studies from 7 countries were included in the analysis. Among 1970 patients, cancer was only found in 22 (0.01 %) cases.

The risk of malignancy after a radiologically proven episode of acute uncomplicated diverticulitis was low. Patients with complicated diverticulitis had a significant risk of colorectal cancer at subsequent colonic evaluation.

A retrospective study of 633 patients with acute diverticulitis diagnosed by CT was published in 2014 [59]. Of 663, 97 underwent emergency resection, whereas 536 were treated conservatively, 394 of whom underwent colonoscopy. The findings showed 17 cancers (2.7 %) in patients with an initial diagnosis of acute diverticulitis. As shown by CT, 16 cancer patients (94 %) had abscess, whereas one patient had pericolic extraluminal air but no abscess. Of the patients with abscess, 11.4 % had cancer mimicking acute diverticulitis. No cancer was found in the patients with uncomplicated diverticulitis.

### Treatment of diffuse peritonitis

Although most patients hospitalized for acute diverticulitis can be managed by non-operative treatment, up to 25 % may require urgent operative intervention [60]. Patients with diffuse peritonitis are typically critical ill patients and require prompt fluid resuscitation, antibiotic administration, and surgery without delay.

Although the absolute prevalence of perforated diverticulitis complicated by generalized peritonitis is low, it has a significant postoperative mortality, regardless of selected surgical strategy.

A critical issue may be the CT presence of distant free air without diffuse fluids (fluids in 2 or >2 abdominal quadrants) because distant pneumoperitoneum is pathognomonic for sigmoid perforation even in absence of CT findings of diffuse peritoneal fluid.

16) Patients with CT findings of distant air without diffuse fluid may be treated by conservative treatment in selected cases. However, there is a risk of treatment failure and emergency surgery may be required. Careful monitoring is mandatory. A CT scan should be repeated early on the basis of the clinical and laboratory evaluation (Recommendation 1 C).
17) If the conservative treatment fails in patients with distant air without diffuse fluid, surgical resection and anastomosis with or without stoma or Hartmann resection is suggested according to the patient clinical conditions and comorbidities (Recommendation 1 B).

Although CT findings of distant free air (a known predictor of failure of non-operative treatment [25]), Dharmarajan et al. [61] described a high success rate for non-operative management in patients with acute diverticulitis and a pneumoperitoneum, excluding those with hemodynamic instability. Sallinen et al. [62] reported results of non-operative management in patients with CT verified extra-luminal air. The study showed that non operative treatment was feasible therapy only for hemodynamic stable patients with pericolic extraluminal air or with small amount of distant intraperitoneal air in the absence of clinical diffuse peritonitis or fluid in the fossa Douglas. Occurrence of large amount of distant intraperitoneal air or distant retroperitoneal air even in the absence of clinical generalised peritonitis was associated with high failure rate (57 %–60 %) of non-operative management. Moreover nearly 60 % patients with distant intraperitoneal air were primary treated by surgery.

Highly selected group of patients at this stage may be treated by conservative treatment. However it may be associated with failure and a careful clinical and CT monitoring is mandatory [7]. Suggested intervention for patients at this stage should be surgical resection and anastomosis with or without stoma in stable patients without co-morbidities and Hartmann resection in unstable patients or in patients with multiple co-morbidities [7].

18) Laparoscopic peritoneal lavage and drainage should not be considered the treatment of choice in patients with generalized peritonitis. (Recommendation 1 A).

A conservative approach using laparoscopic peritoneal lavage and drainage has been debated in recent years as an alternative to colonic resection [63]. It can potentially avoid a stoma in patients with diffuse peritonitis. It consists of the laparoscopic aspiration of pus followed by abdominal lavage and the placement of abdominal drains, which remain for many days after the procedure.

In 2013 a Dutch retrospective analysis of 38 patients [64] treated by laparoscopic lavage was published highlighting some doubts about this procedure to treat critically ill patients. In seven patients laparoscopic lavage did not control abdominal sepsis, two died of multiple organ failure and five required further surgical interventions (three Hartmann resection, one diverting stoma and one perforation closure). One of these died from aspiration and the remaining four experienced prolonged complicated recovery. Multiple co-morbidities, immunosuppression, a high CRP level and/or a high Mannheim Peritonitis Index were also predictors of a high risk of failure. The authors concluded that patient selection was of utmost importance and identification of an overt sigmoid perforation is of critical importance.

Great debate is still open on this topic, mainly due to the discrepancy and sometime disappointing results of the latest prospective trials such as SCANDIV, Ladies, and DILALA trials [65–67]

In 2014 the first results from the randomized controlled trial DILALA were published [65]. Initial diagnostic laparoscopy showing Hinchey III disease was followed by randomization between laparoscopic lavage and colon resection and stoma. Morbidity and mortality after laparoscopic lavage did not differ when compared with the Hartmann procedure. Laparoscopic lavage resulted in shorter operating time, shorter time in the recovery unit, and shorter hospital stay with the avoidance of a stoma. In this trial, laparoscopic lavage as treatment for patients with perforated diverticulitis Hinchey III disease was feasible and safe in the short-term.

In 2015 the results of SCANDV study were published [66]. Among patients with likely perforated diverticulitis and undergoing emergency surgery, the use of laparoscopic lavage vs primary resection did not reduce severe postoperative complications and led to worse outcomes in secondary end points. These findings do not support laparoscopic lavage for treatment of perforated diverticulitis. In the same year, the result of LADIES Study was published. This showed that laparoscopic lavage was not superior to sigmoidectomy for the treatment of purulent perforated diverticulitis [67].

19) Hartmann resection is still advised for managing diffuse peritonitis in critically ill patients and in patients with multiple comorbidities. However in clinically stable patients with no co-morbidities primary resection with anastomosis with or without a diverting stoma may be performed (Recommendation 1 B).

Hartmann resection has been considered the procedure of choice in patients with generalized peritonitis and remains a safe technique for emergency colectomy in diverticular peritonitis, especially in critically ill patients and in patients with multiple co-morbidities. However restoration of bowel continuity after a Hartmann procedure has been associated with significant morbidity [68]. Many patients cannot undergo reversal surgery due to comorbidities; therefore, they remain with a permanent stoma [69].

Common use of Hartmann’s resection in treating diverticular perforation worldwide is confirmed by a recent Australian study analyzing administrative data of patients with acute diverticulitis admitted, from 2009 to 2013, in eight tertiary referral centres with specialist colorectal services [70]. Among 2829 emergency admissions for AD across 4 years in eight hospitals, 724 were for complicated acute diverticulitis. The emergency operative intervention rate was 10.4 %, with one third of the admissions for complicated diverticulitis having an operation. Hartmann's procedure was the most commonly performed emergency operation, accounting for 72 % of resections.

Another population-based retrospective cohort study using administrative discharge data, conducted in Ontario (Canada) was published in 2014 [71]. Among 18,543 patients hospitalized with a first episode of diverticulitis, from 2002 to 2012, 3873 underwent emergency surgery. The use of laparoscopy increased (9 % to 18 %, *p* <0.001), whereas the use of the Hartmann’s procedure remained unchanged (64 %) and was the most frequently used urgent operative approach in patients with complicated AD.

In recent years, some authors have reported the role of primary resection and anastomosis with or without a diverting stoma, in the treatment of diverticulitis, even in the presence of diffuse peritonitis [72]. The decision regarding the surgical choice in patients with diffuse peritonitis is generally left to the judgment of the surgeon, who takes into account the clinical condition and the comorbidities of the patient. Studies comparing mortality and morbidity of Hartmann’s procedure versus primary anastomosis did not show any significant differences. However, most studies had relevant selection bias as demonstrated by four systematic reviews [73–76].

A comparison of primary resection and anastomosis (PRA) with or without defunctioning stoma to Hartmann's procedure (HP) as the optimal operative strategy for patients presenting with Hinchey stage III-IV, was published by Constantinides et al. [76]. A total of 135 PRA, 126 primary anastomoses with defunctioning stoma (PADS), and 6619 Hartmann's procedures (HP) were considered in the study. Morbidity and mortality was 55 % and 30 % for PRA, 40 % and 25 % for PADS, and 35 % and 20 % for HP. Stomas remained permanent in 27 % of HP and in 8 % of PADS. The authors concluded that primary anastomosis with defunctioning stoma may be the optimal strategy for selected patients with diverticular peritonitis and may represent a good compromise between postoperative adverse events, long-term quality of life and risk of permanent stoma.

A small randomized trial of primary anastomosis with ileostomy vs Hartmann’s procedure in patients with diffuse diverticular peritonitis was published by Oberkofler et al. in 2012 [77]. Sixty-two patients with acute left-sided colonic perforation (Hinchey III and IV) from 4 centers were randomized to Hartmann procedure (*n* = 30) and to primary anastomoses with diverting ileostomy (*n* = 32). A planned stoma reversal operation was performed after 3 months in both groups. The study reported no difference in initial mortality and morbidity (mortality 13 % vs 9 % and morbidity 67 % vs 75 % in Hartmann procedure vs primary anastomosis), but a reduction in length of stay, lower costs, fewer serious complications and greater stoma reversal rates in the primary anastomosis group.

20) Emergency laparoscopic sigmoidectomy for the treatment of perforated diverticulitis with generalised peritonitis is feasible in selected patients provided they are handled by experienced hands (Recommendation 2 C).

Laparoscopic sigmoidectomy for diverticulitis has initially been confined to the elective setting however in stable patients, laparoscopic sigmoidectomy may be feasible in purulent and fecal diverticular peritonitis in the emergency setting. In 2015 a systematic review on laparoscopic sigmoidectomy for diverticulitis in the emergency setting was published [78].

The review included 4 case series and one cohort study (total of 104 patients) out of 1,706 references. Hartmann's procedure (HP) was performed in 84 patients and primary anastomosis in 20. The mean operating time varied between 115 and 200 min. The conversion rate varied from 0 to 19 %. The mean length of hospital stay ranged between 6 and 16 days. Surgical re-intervention was necessary in 2 patients. In 20 patients operated upon without defunctioning ileostomy, no anastomotic leakage was reported. Three patients died during the postoperative period. Stoma reversal after HP was performed in 60 out of 79 evaluable patients (76 %).

These guidelines are limited by the low quality evidence which showed that emergency laparoscopic sigmoidectomy for the treatment of perforated diverticulitis with generalised peritonitis is feasible. These studies occurred in selected patients and in experienced units and are not generalizable to all centers. High-quality prospective or randomised studies are needed to demonstrate benefits of acute laparoscopic sigmoidectomy compared to open sigmoidectomy for perforated diverticulitis.

21) Damage control surgery strategy may be suggested for clinically unstable patients with diverticular peritonitis (severe sepsis/septic shock) (Recommendation 1 B).

In an unstable patients with diverticular peritonitis ‘damage control surgery’ has become a valuable technique in the last years [79]. Damage control with lavage, limited bowel resection, laparostomy, and scheduled second-look operation represents a feasible strategy in patients with perforated diverticulitis (Hinchey III and IV) to enhance sepsis control and improve rate of anastomosis.

Generalized diverticular peritonitis is a life-threatening condition requiring prompt emergency operation. To improve outcome and reduce the rate of colostomy formation, a new algorithm with damage control operation, lavage, limited closure of perforation, and second look surgery to restore intestinal continuity was developed in recent years [80, 81]. Critically ill patients (patients with severe sepsis and septic shock) present with hypotension and myocardial depression, combined with coagulopathy. These patients, who are hemodynamically unstable, are not optimal candidates for immediate complex operative interventions. After initial surgery, which should be limited to source control e.g. primary closure of the perforation, the patient is taken to the ICU for physiologic optimization. This strategy may delay bowel anastomosis [81] and potentially avoid stoma formation.

In the setting of diverticulitis several reports (with low level of evidence) were published. In 2010 a prospective observational study was published by Kafka-Ritsch et al. [79]. A total of 51 patients (28 female, 55 %) with a median age of 69 (range) 28–87 years, with perforated diverticulitis Hinchey III (n = 40, 78 %) or Hinchey IV (*n* = 11, 22 %) were prospectively enrolled in the study. Patients were initially managed with limited resection, lavage and temporary abdominal closure followed by second, reconstructive operation 24–48 h later, which are supervised by a colorectal surgeon. Bowel continuity was restored in 38 (84 %) patients, of which four were protected by a loop ileostomy. Five anastomotic leaks (13 %) were encountered requiring loop ileostomy in two patients or Hartmann procedure in remaining three patients. The overall mortality rate was 9.8 % and 35/46 (76 %) of the surviving patients left the hospital with reconstructed colon continuity. Fascial closure was achieved in all patients.

### Planning elective surgery

22) Patient-related factors and not number of previous episodes of diverticulitis, should be considered in planning elective sigmoid resection in patients with ALCD treated conservatively (Recommendation 1 C).
23) After a conservatively treated episode of ALCD an elective sigmoid resection should be planned in high-risk patients, such as immunocompromised patients (Recommendation 1 C).

Recurrence of acute diverticulitis is lower than previously thought. It has been frequently reported that about one third of all patients with acute diverticulitis will have a recurrent attack, often within one year [82, 83]. Recurrence after an uncomplicated episode of diverticulitis, however, has recently been shown to be much lower, with one prospective study reporting a recurrence of only 1.7 % over five years of follow up [84, 85].

In 2014, a systematic review of studies reviewing the diagnosis and management of chronic and recurrent diverticulitis (from studies published between January 2000 to March 2013) was published [86]. The 68 studies included were almost exclusively observational and had limited certainty of treatment effect. The authors found that complicated recurrence after recovery from an uncomplicated episode of diverticulitis was rare (<5 %) and that age at onset younger than 50 years and 2 or more recurrences did not increase the risk of complications.

The authors concluded that the indication for elective colectomy following 2 episodes of diverticulitis is no longer accepted. Indication to colectomy should be made based on consideration of the risks of recurrent diverticulitis, the morbidity of surgery, ongoing symptoms, the complexity of disease, and operative risk.

Clear indications for elective sigmoid resections are complaints of stenosis, fistulas, or recurrent diverticular bleeding. Furthermore, an elective sigmoid resection should be justified in high-risk patients such as immunocompromised patients, after a conservatively treated episode of diverticulitis [87].

### Antimicrobial therapy

24) The empirically designed antimicrobial regimen depends on the underlying clinical condition of the patient, the pathogens presumed to be involved, and the risk factors indicative of major resistance patterns (Recommendation 1 C).
25) Although discontinuation of antimicrobial treatment should be based on clinical and laboratory criteria, a 4–6 days period of post-operative antimicrobial therapy in complicated ALCD is suggested if source control has been adequate (Recommendation 1 A).

Antimicrobial therapy plays an important role in the management of complicated acute diverticulitis. It is typically empiric antibiotic treatment. The empirically designed antimicrobial regimen depends on the underlying severity of infection, the pathogens presumed to be involved, and the risk factors indicative of major resistance patterns [41]. Several recommendations have been recently published in literature [41, 88] in the setting of intra-abdominal infections. However consideration of local epidemiological data and regional resistance profiles is essential for antibiotic selection.

Considering intestinal microbiota of large bowel acute diverticulitis requires antimicrobial coverage for gram-positive and gram-negative bacteria, as well as for anaerobes. Most of the complicated acute diverticulitis is community acquired infection. The main resistance threat in intra-abdominal infections is posed by Extended-Spectrum Beta-Lactamase (ESBL)-producing *Enterobacteriaceae,* which are becoming increasingly common in community-acquired infections worldwide [41]. The most significant risk factors for ESBL producing infection include prior exposure to antibiotics and comorbidities requiring concurrent antibiotic therapy [41]. Anti-ESBL-producer coverage should be warranted for patients with these risk factors. Although discontinuation of antimicrobial treatment should be based on clinical and laboratory criteria such as fever and markers of inflammation, a period of 4–6 days for adult patients is generally sufficient to treat in patients with acute diverticulitis who have been treated with proper source control and prompt surgical intervention [41, 88].

The recent prospective trial by Sawyer et al. demonstrated that in patients with complicated intra-abdominal infections undergoing an adequate source-control procedure, the outcomes after approximately 4 days fixed-duration antibiotic therapy were similar to those after a longer course of antibiotics that extended until after the resolution of physiological abnormalities [89]. Patients who have signs of sepsis beyond 5 to 7 days of antibiotic treatment warrant aggressive diagnostic investigation to determine if an ongoing uncontrolled source of infection exists.

## Conclusions

In appendix 1 WSES recommendations for the management of ALCD are illustrated.

## Abbreviations

ALCD, acute left sided colonic diverticulitis; CT, computed tomography; US, ultrasound

## Appendix 1

1. An accurate assessment of the patients using clinical signs, laboratory inflammation markers and radiological findings is recommended to identify the best treatment for each patient with ALCD (Recommendation 1 C).
2. The clinical diagnosis of ALCD alone is not sufficiently accurate for patients with suspected diverticulitis (Recommendation 1 C).
3. Pain in the lower left abdomen on physical examination and C-reactive protein (CRP) 50 mg/l or more suggests a diagnosis of ALCD (Recommendation 1 C).
4. Computed tomography (CT) scan of the abdomen and pelvis is indicated for all patients with suspected ALCD. It has high sensitivity and specificity and can assess the severity of diverticulitis guiding clinicians in planning treatment (Recommendation 1 C).
5. Ultrasound (US) may be a useful alternative in the initial evaluation of patients with suspected ALCD. It has wide availability and easy accessibility. It may have satisfactory sensitivity and specificity when performed by an expert operator. A step-up approach with CT performed after an inconclusive or negative US may be a safe approach for patients suspected of acute diverticulitis (Recommendation 1 C).
6. Immunosuppression can increase the complication rate in patients with ALCD. Elective sigmoid resection after an episode of ALCD should be recommended in immunocompromised patients (Recommendation 1 C).
7. Antimicrobial therapy can be avoided in immunocompetent patients with uncomplicated diverticulitis without systemic manifestations of infection (Recommendation 1 A).
8. If patients need antimicrobial therapy, oral administration may be acceptable (Recommendation 1 B).
9. Outpatient management is suggested for patients with uncomplicated acute diverticulitis, with no comorbidities. These patients should be clinically monitored as outpatients and re-evaluated within 7 days to assess for resolution of the inflammatory processes. Earlier revaluation is necessary if the clinical condition deteriorates (Recommendation 1 B).
10. Patients with CT findings of pericolic air or small fluid collection should be managed by antimicrobial therapy. (Recommendation 1 C).
11. Patients with small diverticular abscesses (<4-5 cm) may be treated by antibiotics alone (Recommendation 1 C).
12. Patients with large abscesses (>4–5 cm) can best be treated by percutaneous drainage combined with antibiotic treatment (Recommendation 1 C).
13. Whenever percutaneous drainage of the abscess is not feasible or not available, based on the clinical conditions patients with large abscesses can be initially treated by antibiotic therapy alone. However careful clinical monitoring is mandatory. (Recommendation 1 C).
14. In patients with diverticular abscesses treated conservatively early colonic evaluation (4–6 weeks) should be planned (Recommendation 1 C).
15. In patients with CT-proven uncomplicated diverticulitis treated conservatively (without other risk factors) early follow-up colonoscopy is not required. Patients aged 50 years or older should participate in colorectal cancer screening programs (Recommendation 1 C).
16. Patients with CT findings of distant air without diffuse fluid may be treated by conservative treatment in selected cases. However, there is a risk of treatment failure and emergency surgery may be required. Careful monitoring is mandatory. A CT scan should be repeated early on the basis of the clinical and laboratory evaluation (Recommendation 1 C).
17. If the conservative treatment fails in patients with distant air without diffuse fluid, surgical resection and anastomosis with or without stoma or Hartmann resection is suggested according to the patient clinical conditions and comorbidities (Recommendation 1 B).
18. Laparoscopic peritoneal lavage and drainage should not be considered the treatment of choice in patients with generalized peritonitis. (Recommendation 1 A).
19. Hartmann resection is still advised for managing diffuse peritonitis in critically ill patients and in patients with multiple comorbidities. However in clinically stable patients with no co-morbidities primary resection with anastomosis with or without a diverting stoma may be performed (Recommendation 1 B).
20. Emergency laparoscopic sigmoidectomy for the treatment of perforated diverticulitis with generalised peritonitis is feasible in selected patients provided they are handled by experienced hands (Recommendation 2 C).
21. Damage control surgery strategy may be suggested for clinically unstable patients with diverticular peritonitis (severe sepsis/septic shock) (Recommendation 1 B).
22. Patient-related factors and not number of previous episodes of diverticulitis, should be considered in planning elective sigmoid resection in patients with ALCD treated conservatively (Recommendation 1 C).
23. After a conservatively treated episode of ALCD an elective sigmoid resection should be planned in high-risk patients, such as immunocompromised patients (Recommendation 1 C).
24. The empirically designed antimicrobial regimen depends on the underlying clinical condition of the patient, the pathogens presumed to be involved, and the risk factors indicative of major resistance patterns (Recommendation 1 C).
25. Although discontinuation of antimicrobial treatment should be based on clinical and laboratory criteria, a 4–6 days period of post-operative antimicrobial therapy in complicated ALCD is suggested if source control has been adequate (Recommendation 1 A).
